# HIV-1 Entry and Membrane Fusion Inhibitors

**DOI:** 10.3390/v13050735

**Published:** 2021-04-23

**Authors:** Tianshu Xiao, Yongfei Cai, Bing Chen

**Affiliations:** 1 Division of Molecular Medicine, Boston Children’s Hospital, Boston, MA 02115, USA; 2 Department of Pediatrics, Harvard Medical School, Blackfan Street, Boston, MA 02115, USA

**Keywords:** HIV, envelope glycoprotein, viral entry, membrane fusion, fusion inhibitor

## Abstract

HIV-1 (human immunodeficiency virus type 1) infection begins with the attachment of the virion to a host cell by its envelope glycoprotein (Env), which subsequently induces fusion of viral and cell membranes to allow viral entry. Upon binding to primary receptor CD4 and coreceptor (e.g., chemokine receptor CCR5 or CXCR4), Env undergoes large conformational changes and unleashes its fusogenic potential to drive the membrane fusion. The structural biology of HIV-1 Env and its complexes with the cellular receptors not only has advanced our knowledge of the molecular mechanism of how HIV-1 enters the host cells but also provided a structural basis for the rational design of fusion inhibitors as potential antiviral therapeutics. In this review, we summarize our latest understanding of the HIV-1 membrane fusion process and discuss related therapeutic strategies to block viral entry.

## 1. HIV-1 Entry 

**Figure 1:**
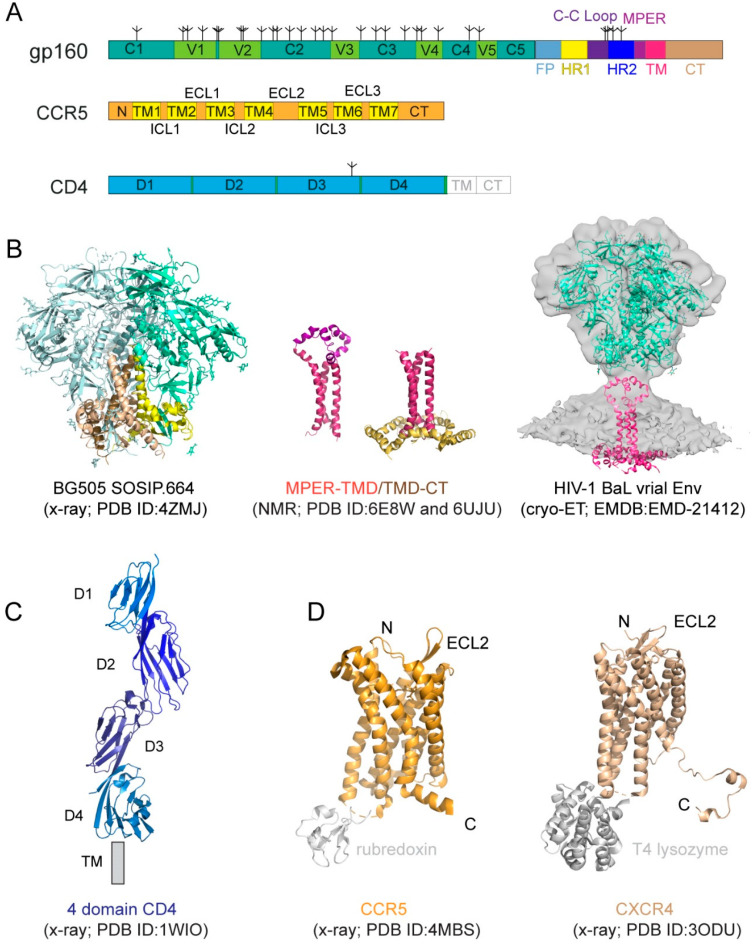
HIV-1 (human immunodeficiency virus type 1) envelope glycoprotein and its receptors. (**A**) The full-length HIV-1 Env, gp160. Segments of gp120 and gp41 include: C1–C5, conserved regions 1–5; V1–V5, variable regions 1–5; F, fusion peptide; HR1, heptad repeat 1; C-C loop, the immunodominant loop with a conserved disulfide; HR2, heptad repeat 2; MPER, membrane proximal external region; TM, transmembrane anchor; CT, cytoplasmic tail; tree-like symbols, glycans. Those for CCR5 include: N, N-terminus; TM1-7, transmembrane helices 1–7; ECL1-3, extracellular loop 1–3; ICL3, intracellular loop 1–3; and CT, cytoplasmic tail. For CD4, they are: D1–4, immunoglobulin (Ig) domain 1–4; TM and CT. (**B**) Structures of HIV-1 Env. The crystal structure of the unliganded HIV-1 BG505 SOSIP.664 Env trimer (pdb ID: 4ZMJ; [11]) that lacks the MPER, TMD, and CT is shown in the ribbon diagram with gp120 in cyan and gp41 in yellow. Structures of the MPER-TMD and TMD-CT reconstituted in bicelles that mimic lipid bilayer determined by NMR (pdb ID: 6E8W; [12]; pdb ID: 6UJU; [13]). The MPER is in magenta, the TMD in dark red, and the CT in gold. The EM density in gray is 3D reconstruction of the unliganded HIV-1 BaL Env spike on the surface of virion by cryo-electron tomography (EMDB ID: EMD-21412). (**C**) Crystal structure of soluble four domain CD4 (pdb ID: 1WIO; [14]). D1-D4 and the location of the transmembrane segment (TM) are indicated. (**D**) Crystal structure of a modified CCR5 in complex with Maraviroc (pdb ID: 4MBS; [15]). CCR5 is shown in the ribbon diagram in brown, the internally fused rubredoxin and the ligand in gray. N-terminus (N), C-terminus (**C**), and the second extracellular loop (ECL2) are indicated. Crystal structure of an engineered CXCR4 in complex with a viral chemokine antagonist IT1t (pdb ID: 3ODU; [16]). CXCR4 is shown in brown, the fused T4 lysozyme and the ligand in gray.

The strategy that enveloped viruses, such as HIV-1 (human immunodeficiency virus type 1), use to gain entry into their host cells is membrane fusion, which is an energetically favorable process but with high kinetic barriers [1,2]. Virus-encoded fusion proteins are catalysts and undergo structural rearrangements from a high-energy, metastable prefusion conformation to a low-energy, stable postfusion conformation, providing free energy for overcoming these kinetic barriers [3,4,5]. In the case of HIV-1, its envelope glycoprotein (Env) functions as the fusion protein. The Env protein is synthesized as a precursor, gp160 (for glycoprotein with an apparent molecular weight of 160 kDa; Figure 1A), which forms a trimer (gp160)_3_ and is then cleaved by a cellular furin-like protease into two noncovalently associated subunits: the receptor-binding subunit gp120 and the fusion subunit gp41 [6]. Three copies of each subunit constitute the mature envelope spike (gp120/gp41)_3_. It is generally believed that sequential binding of gp120 to primary receptor CD4 and coreceptor (e.g., chemokine receptor CCR5 or CXCR4) initiates a cascade of refolding events in gp41 that drive the membrane fusion process [7,8]. The mature Env spikes are also the sole antigens on the surface of virion and induce strong immune responses in infected individuals [9,10]. Not surprisingly, HIV-1 Env is a critical target for the development of both vaccines and therapeutics against the virus. Recent advances in the structural biology of HIV-1 Env and its complexes with the host receptors, as well as in the design of novel fusion inhibitors, have provided new insights into HIV-1 entry and its inhibition. In this review, we summarize our latest understanding of the membrane fusion catalyzed by HIV-1 Env and discuss related therapeutic strategies to block viral entry.

## 2. Structures of HIV-1 Env and Cellular Receptors

### 2.1. HIV-1 Env

The HIV-1 Env is a heavily glycosylated type I membrane protein with five conserved regions 1–5 (C1-C5) and five variable regions 1–5 (V1-V5) in gp120; and various segments in gp41 including a hydrophobic fusion peptide (FP), two heptad-repeat regions (HR1 and HR2), a disulfide-bridged loop (C-C loop), a tryptophan-rich membrane proximal external region (MPER), a transmembrane segment (TM) and a cytoplasmic tail (CT) (Figure 1A). The protein has been historically a very challenging target for structural analysis due to technical difficulties associated with large membrane-bound glycoproteins. Nevertheless, a truncated version of gp120, named ‘gp120 core’, with V1-V3 and terminal segments deleted, was crystallized in two forms: a deglycosylated one in complex with CD4 and a CD4-induced antibody for HIV-1 [17] and an unliganded and fully glycosylated one for closely related simian immunodeficiency virus (SIV) [18], producing structures that gave us the first glimpse of gp120 folding and its interaction with CD4. Likewise, the structure of a gp41 fragment of HR1 and HR2 has been solved by X-ray crystallography and nuclear magnetic resonance (NMR) [7,8,19,20,21], revealing the postfusion conformation of gp41 as a six-helix bundle, in which the HR1 and HR2 helices are arranged into a trimer of hairpins. 

The first breakthrough on high-resolution structures of the Env trimer only came more than a decade later from a designed soluble construct, termed ‘SOSIP’, with stabilizing modifications (i.e., a disulfide bond between gp120 and gp41, an I559P substitution in gp41, and a truncation at residue 664 deleting the MPER; [22]) by both cryogenic electron microscopy (cryo-EM) and X-ray crystallography [23,24,25] (Figure 1B). Subsequently, the structure of a detergent-solubilized Env trimer without the CT and SOSIP modifications was determined in complex with neutralizing antibodies by cryo-EM [26]. More recently, the cryo-EM structures of two full-length HIV-1 Env constructs purified in detergent have also been reported [27,28]. These trimer structures have shown that the prefusion gp41 adopts a drastically different conformation from the postfusion six-helix bundle structure and provided much-needed insights on Env structure and its conformational changes. The MPER, TMD, and CT are all disordered in these structures, however, highlighting the important role of the lipid bilayer in stabilizing the structure of these regions. An attempt to determine the structure of the missing regions using a full-length Env reconstituted in lipid nanodiscs did not yield much additional high-resolution information [29]. In addition, cryo-electron tomography (cryo-ET) has been used to study the structures of Env trimer on the surface of both HIV and SIV chemically inactivated virions, leading to reconstructions at a low resolution (~20 Å) during early days [30,31,32,33], and a more recent one at ~10Å resolution (Figure 1B; [34]), but the regions near the membrane remain difficult to resolve.

Recent data indicate that the membrane-related components of HIV-1 Env, including the MPER, TM domain (TMD), and CT, influence the stability and antigenicity of the Env ectodomain, as well as cell–cell fusion and viral infection [12,35,36,37,38,39], in agreement with their conserved features. For example, the MPER has been studied extensively because it contains epitopes recognized by a group of broadly neutralizing antibodies [40,41,42,43,44]. The TMD has a ‘GXXXG’ motif and a highly conserved positively charged residue (Lys or Arg). The CT includes the Kennedy sequence, three conserved amphipathic α-helices segments referred to as a lentiviral lytic peptide (LLPs: LLP1, LLP2, and LLP3) [45,46,47]. Truncation of the CT of the full-length HIV-1 Envs has minimal impact on their fusogenic activity, but it has an unexpectedly large impact on the antigenic structure of the ectodomain [35]. Some other studies showed that the CT modifications had little effect on the Env antigenicity for certain HIV-1 isolates [48,49,50]. Nevertheless, structural studies in the context of a lipid bilayer appear to support crosstalk between the CT and the ectodomain.

NMR spectroscopy and isotopic labeling techniques enable the structural determination of peptides and small proteins at high resolutions, particularly for those with some flexibility. 2D Transverse relaxation-optimized spectroscopy (2D-TROSY) combined with 3D spectroscopies and selective deuterium-labeling methods allows structural characterization of oligomeric membrane proteins in micelles or bicelles. Moreover, functional dynamics and protein–protein/ligand interactions can be quantitatively measured by NMR techniques at the residue level. Thus, NMR spectroscopy is a powerful approach suitable for structural analysis of the MPER, TMD, and CT regions of gp41 [51]. For instance, the structure of the TMD reconstituted in bicelles mimicking a lipid bilayer was first determined by NMR [36] (Figure 1B). The TMD forms a well-ordered trimer, and that mutational changes disrupting the TMD trimer alter antibody sensitivity of the ectodomain, suggesting that the TMD contributes to Env stability and antigenicity. Moreover, although previous studies reported that the MPER might be buried in the viral membrane [52,53,54], the NMR structure that contains both the MPER and TMD in the bicelle system has shown that the MPER forms a well-ordered trimeric assembly, not buried in the membrane [12] (Figure 1B). The MPER mutations can destabilize the Env ectodomain and shift it towards an open conformation, suggesting that the MPER is a control relay that modulates open and closed states of the Env trimer. Furthermore, a third NMR structure containing the TMD and part of CT in bicelles has been reported recently [13] (Figure 1B), revealing that the CT folds into amphipathic helices, attached to the bilayer surface, wrapping around the C-terminal end of the TMD and thereby forming a support baseplate for the entire Env. Mutational data have also confirmed that altering the CT-TMD packing interface affects the antigenicity of the full-length Env trimer. These results support the model of a dynamic coupling across the TMD between the CT and ectodomain. Nevertheless, a high-resolution structure of the native, full-length HIV-1 Env in the membrane is still required for a full understanding of its structure and function.

### 2.2. Primary Receptor CD4

CD4 (cluster of differentiation 4) is a membrane-bound glycoprotein found on the surface of immune cells and normally functions as a coreceptor for enhancing T-cell receptor-mediated signaling. CD4 contains four immunoglobulin-like extracellular domains, D1–D4 (Figure 1A), exposed on the cell surface. It was shown to be the primary HIV-1 receptor shortly after the discovery of the HIV virus [55,56,57]. The structure of CD4 alone or in complex with gp120 core has been determined by X-ray crystallography [14,17,58,59,60] (Figure 1C).

### 2.3. Coreceptor

CD4 alone was not sufficient to support HIV-1 infection, leading to intensive search and subsequent identification of CXCR4 and CCR5, the seven-transmembrane (7TM) chemokine receptors, as the coreceptor for the virus [61,62,63,64,65,66]. Coreceptor usage is the primary determinant for viral tropism [67], as those that use CCR5 (R5 viruses) are the dominant form during viral transmission, and others using CXCR4 (X4 viruses) or both (dual-tropic; R5/X4 viruses) emerge mainly during disease progression [68,69,70,71]. Both CCR5 and CXCR4 have a core structure formed by 7TM helices, decorated by an N-terminal segment and three extracellular loops (ECL) exposed outside of the cell, as well as three intracellular loops (ICL), and a cytoplasmic C-terminal tail on the opposite side of the membrane (Figure 1A). A C-terminally truncated CXCR4 construct with stabilizing mutations, and a T4 lysozyme fusion in complex with different ligands, and a similarly modified CCR5 construct containing a rubredoxin fusion in complex with the anti-HIV drug, Maraviroc, have been crystallized, yielding high-resolution structures [15,16,72] (Figure 1D), with a typical 7TM helical bundle topology typically seen for other GPCRs (Figure 1C; [73]). A two-site model has been proposed for their ligand interactions [74], as the N-terminal segment of CXCR4 or CCR5 forms the chemokine recognition site 1 (CRS1) to bind the globular core domain of chemokine, while their TM helices make up the chemokine recognition site 2 (CRS2) to interact with the N-terminus of the chemokine. These structures have revealed the general architecture of these chemokine receptors and their interactions with the ligands [15,16,72,75], but they did not provide many of the molecular details of how they function as HIV-1 coreceptors.

## 3. Molecular Mechanism of HIV-1 Membrane Fusion

### 3.1. Interactions between HIV-1 Env and Cellular Receptors

#### 3.1.1. Interactions between Env and CD4

**Figure 2:**
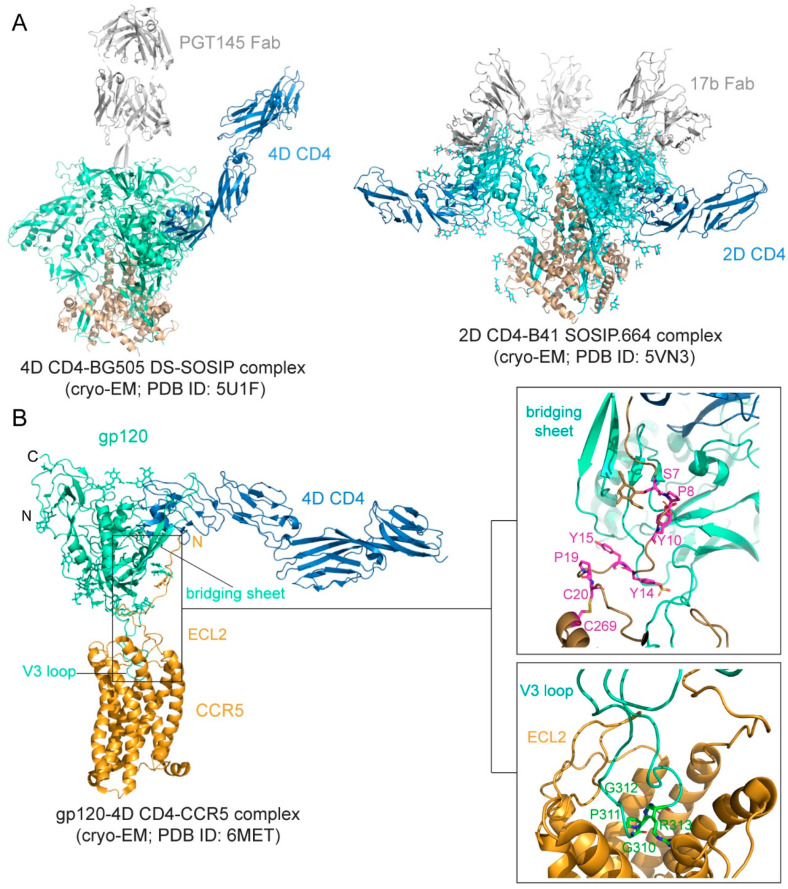
Env-CD4 interaction. (**A**) Left, the cryo-EM (cryogenic electron microscopy) structure BG505 DS-SOSIP.664 Env trimer in complex with 4D CD4 and PGT145 Fab (pdb ID: 5U1F; [82]) is shown with gp120 in cyan, gp41 in brown, CD4 in blue and PGT145 Fab in gray. Right, the cryo-EM structure of B41 SOSIP.664 Env trimer in complex with 2D CD4 and 17b (pdb ID: 5VN3; [80]) is shown with gp120 in cyan, gp41 in brown, CD4 in blue, and 17b Fab in gray. (**B**) Env-CCR5 interaction. Left, overall structure of the 4D CD4-gp120-CCR5 complex (pdb ID: 6MET; [83]) shown in ribbon diagram. N, N-terminus; C, C-terminus; ECL2, extracellular loop 2. V3 loop and the bridging sheet of gp120 are also indicated. Right, close-up views of the interfaces between gp120 and CCR5. The N-terminus of CCR5 is attaching to the surface of the four-stranded bridging β sheet formed by the V1V2 stem and β21–β22 of gp120. Residues Ser7, Pro 8, sulfated Tyr 10, sulfated Tyr14, Tyr15, and Pro19, as well as the disulfide between Cys20 and Cys269 of CCR5 are highlighted in the stick model. The O-linked glycan at Ser7 is also shown. V3 is inserting into the CRS2 of CCR5. The conserved GPGR motif of V3 is highlighted in the stick model, and ECL2 of CCR5 is indicated.

The binding affinity is in the low nM range for soluble CD4 and monomeric gp120 [76,77,78], but it can be ~20 nM for various trimeric forms of soluble Env trimers [78,79]. The binding interface between CD4 and gp120 was first defined in the structure of the gp120 core-CD4 complex [17]. Gp120 core has two separate domains—inner domain and outer domain, and there is also a four-strand β-sheet, named bridging sheet, between the two domains. CD4 interacts with gp120 mainly at the interface between the inner domain and outer domain, inducing the formation of the bridging sheet. The structures of CD4 in complex with Env trimer have been determined using the SOSIP trimer as well. The CD4 bound trimer adopts a more open conformation compared with the unliganded Env SOSIP trimer (Figure 2A). The Env conformational changes include V1–V2 flip, V3 exposure, the bridging sheet formation, and repositioning of the fusion peptide in gp41 [80,81]. Another further constrained trimer, named DS-SOSIP.664, was created by introducing a disulfide bond (201C–433C) into the SOSIP [11]. This trimer binds sCD4 with an asymmetric 1:1 (CD4:trimer) stoichiometry. It appears that a single CD4 molecule is embraced by a quaternary HIV-1 Env surface with the previous defined CD4-binding region in the outer domain of one gp120 protomer and with the second CD4-binding site (CD4-BS2) in the inner domain of a neighboring gp120 protomer (Figure 2A), suggesting that the complex may represent the initial contact of the HIV-1 Env with the CD4 receptor [82].

#### 3.1.2. Interactions between Env and Coreceptor

Preparing stable and homogenous samples of purified CCR5 or CXCR4 has been technically challenging, and various assays have, therefore, been employed to measure the binding affinity for the Env-coreceptor interactions in the presence of soluble CD4 (<10 nM for CCR5; 200–500 nM for CXCR4; [84,85,86,87]). The first structure of a full-length monomeric gp120 in complex with a soluble 4D-CD4 and an unmodified human CCR5 was determined by cryo-EM [83] (Figure 2B), revealing details of the interactions between gp120 and CCR5, largely consistent with the predictions based on previous mutational data [88,89]. The crown of the V3 loop insets into a deep pocket formed by the 7-TM helices of CCR5. The ECL2 forms a semicircular grip and wraps around the V3 loop, making contact with residues from both the V3 stem and crown. The N terminus of CCR5 and the bridging sheet of gp120 make up the second interface between them, in which the N terminal segment of the coreceptor adopts an extended conformation with several sharp turns and latches onto the surface of the bridging sheet (Figure 2B).

### 3.2. Membrane Fusion

**Figure 3:**
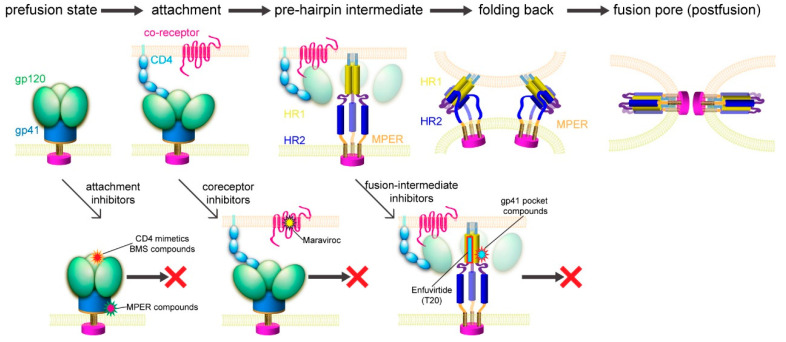
HIV-1 membrane fusion and its inhibition. Top, membrane fusion likely proceeds stepwise as follows. (1) Binding of gp120 to CD4 and a coreceptor allows viral attachment and triggers structural changes in Env. (2) Dissociation of gp120 and insertion of the fusion peptide of gp41 into the target cell membrane leads to the prehairpin intermediate [95]. (3) HR2 folds back onto the inner core of HR1 and brings the two membranes together. (4) A hemifusion stalk forms and resolves into a fusion pore [96]. Bottom, opportunities for fusion inhibitors, including attachment inhibitors targeting the CD4 binding site and the MPER; coreceptor inhibitors; and fusion-intermediate inhibitors.

Previous studies suggested a working model for HIV-1 Env-mediated membrane fusion [7], depicted in Figure 3. Gp120 binding to the receptors induces large structural rearrangements in gp41, which adopts a prefusion conformation within the precursor gp160. The cleavage between gp120 and gp41 primes the protein and makes it metastable with respect to the postfusion conformation. Once triggered, the FP of gp41 translocates and inserts into the target cell membrane. Further refolding of gp41 into a hairpin conformation creates a six-helix bundle structure, thereby placing the FP and TM segments at the same end of the molecule and effectively bringing the two membranes together. Formation of hemifusion stalk and fusion pore ensues membrane fusion and entry of the viral capsid into the target cell. How many receptor molecules are necessary for activating one Env trimer and how many Env trimers are required to induce productive viral entry remain controversial [90]. Early data demonstrated that a fully functional Env trimer could be assembled even with one or more gp120 protomer(s) defective in CD4 binding or coreceptor binding [91], suggesting that a single CD4 or coreceptor is sufficient to trigger the Env trimer. Similarly mixed trimer assays have been used to estimate the number of Env trimers needed for membrane fusion by mathematical modeling, giving a range between 1 and 8 (reviewed in [90]). More recently, an intriguing stoichiometry among Env trimer, CD4, and coreceptor CCR5 or CXCR4 during membrane fusion has been estimated by advanced microscopy and spectroscopy imaging techniques and involves oligomerization of the receptors [92]. smFRET (single molecule fluorescence resonance energy transfer) data also suggest that one CD4 could induce large structural changes within the Env trimer [93]. In another study, fusion inhibitors have been used to trap the pre-hairpin intermediate state of gp41 in an extended conformation at the viral attachment sites, followed by visualization of electron tomography, showing only 2–3 gp41 molecules per attached virion [94]. Since there are only ~14 Env spikes per virion [32], the probability for multiple Env trimers to engage multiple CD4 and coreceptor molecules simultaneously in a synchronized manner would low. Nevertheless, recent progress in the structural biology of HIV-1 Env and its complexes with the cellular receptors has allowed us to fill in additional molecular details of this working model.

The binding of gp120 to CD4 attaches the virus to the surface of the target cell. The SOSIP trimer in a complex of one CD4 molecule shows a possible orientation for CD4 to make the first contact with the Env (Figure 2A). It has been long speculated that the coreceptor binding induces additional structural changes in gp120 that lead to the irreversible refolding of gp41. Unexpectedly, the CD4-gp120-CCR5 structure revealed no obvious allosteric changes in gp120 that could propagate from the CCR5 binding site to gp41, as a comparison of the CD4- and CCR5-bound gp120 and the CD4-bound gp120 showed no major differences in the gp120 core region ([83]; Figure 2B). The only obvious structural changes were the reconfiguration of the V3 loop and flipping back of the N- and C-termini of gp120 near its interface with gp41. In the prefusion structure of the Env trimer [23,24,25,26,80], gp41 folds into a so-called “4-helix collar” with its four helices [25], wrapping around the N- and C-termini of gp120. If gp120 departures, gp41 would be destabilized and likely enter an irreversible refolding process. Partial or complete gp120 dissociation may, therefore, be the crucial “trigger” that prompts a series of refolding events in gp41 and the membrane fusion process. Indeed, CD4 binding causes a large shift of the C-terminal helix away from the gp120 termini, creating a pocket filled by the fusion peptide [80], which normally packs against the gp120 N-terminus. When the fusion peptide flips away from the pocket because of the intrinsic conformational dynamics, it opens up one side of the gp41 grip on the gp120 termini, and the N-terminal segment of gp120 can then bend back to adopt the conformation seen in the CCR5-bound structure. The replacement of the gp120 termini can prevent the fusion peptide from reoccupying the pocket and effectively weaken the gp120-gp41 association. Spontaneous or CD4-induced gp120 dissociation from the Env trimers has been well documented for many HIV-1 isolates [97,98], indicating that gp120 has the tendency to dissociate from gp41 even without a coreceptor. We note that this model is very similar to that proposed for membrane fusion catalyzed by coronavirus spike proteins, in which dissociation of the receptor-binding subunit initiates the irreversible refolding of the metastable fusion subunit, allowing the fusogenic transition to a stable postfusion structure [99,100].

If CCR5 does not induce further structural rearrangements in gp120 to activate gp41, what is a coreceptor needed for then? First, it would be non-productive if gp120 dissociates prematurely in the absence of a coreceptor, because when a virion attaches to the target cell surfaces with the Env trimer forming a complex only with CD4, the distance between the gp41 fusion peptide and the cell surface is not close enough for it to reach the target membrane. Engaging a coreceptor, which is largely embedded in the membrane, will bring the fusion peptide substantially closer [25]. Second, single-molecule force spectroscopy data using infectious virions and live host cells indicated that the Env-CD4 association is unstable and rapidly reversible unless CCR5 binding immediately follows [101,102]. CCR5 is needed merely to stabilize the CD4-induced conformational changes in Env, which are already sufficient to drive membrane fusion. Third, fusion pore formation probably requires 2–3 Env trimers clustered together [26,90,94], also demonstrated for other viral fusion proteins [103]. A stable Env-receptor complex would help synchronize these trimers to undergo the same conformational changes. Thus, a coreceptor probably functions by stabilizing and anchoring the CD4-induced conformation of the Env trimer near the cell membrane to facilitate productive membrane fusion.

## 4. Structure-Guided Development of Membrane Fusion Inhibitors

Although Env has a greater diversity than those virus-encoded enzymes, such as reverse transcriptase or protease, the fusion-promoting conformational changes of Env expose some highly conserved, functionally critical regions and provide attractive targets for developing broad and effective fusion inhibitors (Figure 3). In addition, fusion inhibitors do not need to cross the cell membrane to reach their target; unlike those enzymes, Env has no obvious cellular homologs. It is, therefore, a more likely target for inhibitors with high specificity but minimal side effects.

### 4.1. Protein/Peptide-Based Fusion Inhibitors

#### 4.1.1. Fusion Inhibitors Targeting CD4

A CD4-specific antibody, ibalizumab, which targets an epitope near the D1–D2 junction on the opposite side of the gp120 binding site on CD4 [104], was approved by the United States Food and Drug Administration (USFDA) in March 2018 after demonstrating a significant antiviral potency in patients with multidrug resistance (MDR) in a phase III clinical study [105]. Although ibalizumab remains an expensive treatment and resistance can arise from the reduced expression or loss of N-linked glycosylation sites in V5 of gp120, it is still of great significance for those MDR patients [106].

#### 4.1.2. CD4-Mimetic Inhibitors

Soluble CD4 failed to block infection by HIV-1 clinical isolates, dampening the initial enthusiasm for the decoy-receptor strategy. CD4 binds to gp120 with its residue Phe43 occupying a conserved cavity formed by both the inner and outer domains of gp120 [17,80]. A number of CD4-mimetic miniproteins (miniCD4) have been designed using a scyllatoxin scaffold to present the gp120-binding portion of CD4 to target the CD4 binding site. The best construct, M48U12, has been shown to mimic CD4 and bind to the Phe43 cavity of gp120; it also inhibits the viral infection with a potency in the low nM range [107]. Furthermore, these miniproteins are resistant to acidic pH and high temperature [107], suggesting that they may be promising drug candidates.

#### 4.1.3. Peptide-Based Fusion Inhibitor

The formation of the six-helix bundle structure of gp41 is critical for viral infectivity [5], and there have been many peptide-based inhibitors developed for blocking this step. The synthesized peptides derived from HR1 or HR2 were designed to bind the fusion intermediates of gp41 and prevent the formation of the six-helix bundle [108,109]. In particular, T20/Enfuvirtide, a 36-residue peptide derived from gp41, is the first fusion inhibitor approved by the USFDA [110,111]. Despite its initial success, Enfuvirtide has to be stored at low temperature, freshly reconstituted, and injected subcutaneously twice a day. Moreover, injection site reactions, the rapid emergence of resistant viruses, and the high cost of production have limited its long-term use [112,113,114]. Similar peptide fusion inhibitors, such as T1144 and Sifuvirtide, suffer similar disadvantages [115,116]. Another HR-2 peptide-derived inhibitor, Albuvirtide, also known as FB006M, has received marketing approval in China for the treatment of HIV-1 infection [117]. Conjugation of maleimidopropionic acid to the HR-2 peptide allows its binding to human serum albumin, effectively preventing protease degradation in vivo and leading to a half-life that is ~10-fold longer than that of Enfuvirtide [118].

To further improve the potency of the HR2-based peptides, different strategies have been explored. For example, a so-called ‘M-T hook’ was introduced to the N-terminus of an HR-2 peptide to enhance the stability of the six-helix bundle and anti-HIV-1 potency [119]. Three residues Ile-Asp-Leu (IDL anchor) were added to the C-terminus of another HR2 peptide to target the hydrophobic groove in the HR1 trimer (L544–V549), leading to a ~50-fold increase in the inhibitory activity [120]. Moreover, am-xylene thioether stapling method was used to ‘staple’ two residues separated by three intervening residues (i, i + 4; one helix turn) and to stabilize the helical peptide, preventing protease degradation [121]. In addition to the strategies of modifying the peptides themselves, promoting membrane incorporation by conjugation with lipids has also been considered to increase the effective peptide concentration at the fusion site [122,123]. A series of lipopeptides has been reported with a remarkable inhibitory potency reaching the pM level using different lipids and peptide modifications [124].

### 4.2. Small-Molecule Fusion Inhibitors

Although protein/peptide-based fusion inhibitors generally show great potency with reasonable inhibition breadth and excellent safety profile, there are concerns, including the high cost of production, injection site reaction, pharmacokinetic properties, and potential host immune reactions to foreign peptides. Thus, it is still desirable to develop orally available small-molecule fusion inhibitors, to overcome the limitations of peptide-based drugs.

#### 4.2.1. Fusion Inhibitors Targeting Coreceptors

CCR5 has been explored as an antiviral target because of its essential role in HIV-1 transmission. Individuals who express the CCR5∆32 mutation have been shown to be healthy and almost completely resistant to HIV-1 infection [125,126]. CCR5-specific antibodies downregulate CCR5 expression on CD4+ T lymphocytes and effectively inhibit HIV-1 entry into host cells in vitro [127,128]. Multiple CCR5 antagonists, such as Aplaviroc, Maraviroc, TAK-779, and Vicriviroc, have been developed to block HIV-1 entry [129,130,131,132,133,134]. While the allosteric antagonist Vicriviroc is still in a phase 2 clinical trial and the clinical studies on Aplaviroc were discontinued due to its liver toxicity, Maraviroc has been approved by the USFDA as the first-of-its-kind entry inhibitor. It binds to the cavity within the transmembrane domain of CCR5 and disrupts gp120 binding as a direct competitor [83]. Entry inhibitors targeting CXCR4, such as AMD3100, AMD3465, and AMD070, also show an inhibitory potency at the nM level [135,136,137,138], but they have yet to be approved for clinical use.

#### 4.2.2. Fusion Inhibitors Targeting the CD4 Binding Site

The NBD (N-phenyl-N-piperidin-4-yl-oxalamide analogs) series of CD4-mimetic inhibitors were identified by a high throughput screen [139]. Among them, NBD-556 has been shown to recognize the Phe43 cavity of gp120 and inhibit the virus–cell and cell–cell fusion with an IC50 in a low µM range [139]. Like CD4, NBD-556 binding to gp120 can trigger conformational changes and formation of the CCR5 binding site, and thus enhancing the infection to CD4-/CCR5+ cells. An optimized derivative NBD-11021 exhibited further improved antiviral activity without the enhanced infection to the CD4-/CCR5+ cells [140].

**Figure 4:**
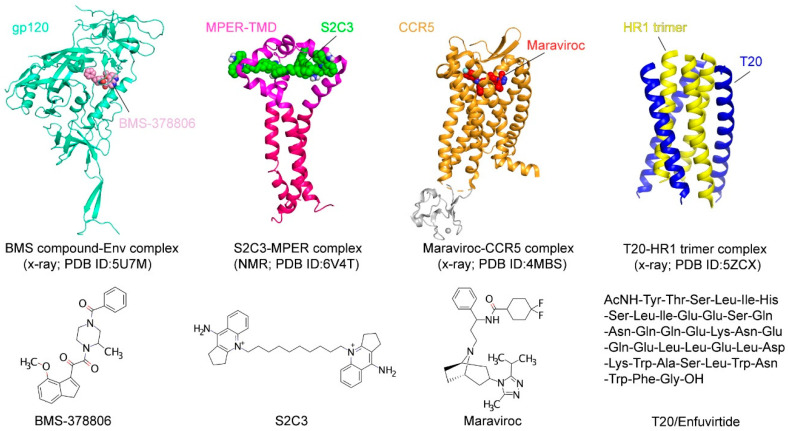
Fusion inhibitors. Various vulnerable sites of HIV-1 entry, including the BMS compound binding site in gp120 (pdb ID: 5U7M; [142]), the MPER (pdb ID: 6V4T; [147]), the CCR5 TM pocket (pdb ID: 4MBS; [15]), and the T20 binding groove on the HR-1 trimer (pdb ID: 5ZCX; [148]), are targeted by different entry/fusion inhibitors, including BMS-378806, S2C3, Maraviroc, and T20/Enfuvirtide.

Bristol–Myers Squibb identified a small-molecule inhibitor in 2003, BMS-806 (also known as BMS-378806), by high-throughput screen using a cell-based assay (Figure 4) [141]. The optimized derivatives, including BMS-488043, BMS-626529, and BMS-663068, showed greater potencies than BMS-806 [142,143,144]. BMS-663068 (Fostemavir) is the first FDA-approved attachment inhibitor for use in combination with other antiretrovirals in highly treatment-experienced patients with multidrug-resistant HIV-1 infection [145]. Although the BMS compounds were thought to target the Phe43 cavity as suggested by mutagenesis studies [146], the crystal structure of the Env-BMS compound complex revealed a different binding pocket (Figure 4), located at the interface between the outer and inner domains of gp120 under the β20–β21 loop [142]. Thus, the BMS compounds affect the local structure near its binding pocket and block the viral attachment allosterically.

#### 4.2.3. Fusion Inhibitors Targeting the Hydrophobic Pocket of gp41

Structural studies of the gp41 postfusion structure revealed a hydrophobic pocket formed by the HR1 helices [149]. ADS-J1 is the first small-molecule inhibitor identified by a computer-aided molecular docking tool to recognize the gp41 pocket with an inhibitory potency of low μM [150]. Another compound, 5M038, was identified from a high throughput screen relying on competition with an HR-2 peptide binding to a designed HR-1 trimer construct (5-helix) [109,151]. 5M038 binding to the hydrophobic pocket was confirmed by the broadened and shifted peaks in the 1H-NMR spectrum. It has a potency at the µM level in protein-based inhibition, cell–cell fusion, and viral infectivity assays [151]. Other potentially pocket-targeting compounds include N-(4-carboxy-3-hydroxyphenyl)-2,5-dimethylpyrrole (NB-2), N-(3-carboxy-4-chloro) phenylpyrrole (NB-64), one NB-64 derivative A12 and NB-293 [152]. However, their mechanisms of action require further validation or investigation.

#### 4.2.4. Fusion Inhibitors Targeting the MPER

The MPER is one of the most conserved regions in gp41 and recognized by several well-characterized broadly neutralizing antibodies, including 2F5, 4E10, Z13e1, 10E8, and DH511 [41,42,43,44]. These antibodies appear to neutralize the virus infection by binding with the prehairpin intermediate state of gp41 with the help of their lipid-binding activity [153,154,155]. Although the great neutralization potencies in vitro, studies involving the passive transfer of 2F5 and 4E10 in HIV-1 infected patients suggest that the antibodies may not be effective in blocking HIV-1 propagation in vivo [156]. It would be desirable to identify small-molecule fusion inhibitors that could target this highly conserved vulnerable site of HIV-1 Env first identified by these neutralizing antibodies.

**Figure 5:**
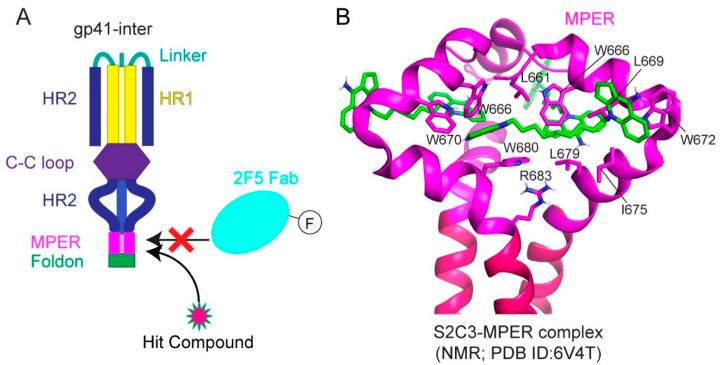
Structure of S2C3 in complex with the MPER-TMD. (**A**) A fluorescence polarization assay for antibody binding to gp41-inter used for the high throughput screen. The binding of a fluoresceinated 2F5 Fab fragment to gp41 can be blocked by hit compounds targeting the MPER by direct competition. F, FITC. (**B**) Close-up views of the hydrophobic binding pocket of S2C3 formed by residues in the MPER in ribbon diagram (pdb ID: 6V4T; [147]). The MPER is in magenta and S2C3 in green. Residues forming the S2C3 binding pocket are indicated.

Indeed, small-molecule compounds were found through a high-throughput screen guided by the neutralizing antibody 2F5 (Figure 5A; [147]). A fluorescence polarization assay was developed based on the 2F5 binding to gp41-inter, an engineered protein designed to mimic the intermediate gp41 (Figure 5A) to identify the small molecules selectively capture MPER and mimic 2F5 to disrupt membrane fusion. One hit compound, dequalinium, was validated by binding and cell–cell fusion assays, with an inhibitory potency of 13.8 μM [147]. Additional dequalinium derivatives were tested, leading to a more potent inhibitor S2C3 than dequalinium, effective against a broad spectrum of HIV-1, HIV-2, and SIV strains with an IC50 of 2–20 μM [147]. Titration of S2C3 to the MPER-TMD reconstituted in bicelles confirmed its interaction with the MPER. The NMR structure of the MPER-TMD in complex with S2C3 revealed a hydrophobic binding pocket formed by the MPER residues in two adjacent protomers (Figure 5B; [147]). Moreover, S2C3 blocks the soluble CD4 binding to the intact Env on the cell surfaces by locking the prefusion conformation and preventing CD4-induced conformational change. The discovery of a small-molecule binding site in the MPER drastically expands the potential medical relevance of this previously recognized vaccine target. Because of their modest potency, dequalinium and its more potent derivatives, such as S2C3, are only the first steps toward a useful anti-HIV-1 drug. The high-resolution structural information can motivate additional HTS campaigns and computational searches to identify more leads for drug candidates suitable for preclinical and clinical investigations.

Finally, the antibody-based screening strategy for drug discovery should be applicable to many other human diseases. Monoclonal antibodies have been used as therapeutics because they can specifically target functional sites of key proteins in various disease-related pathways, but they suffer from drawbacks, including a high cost for production, inability to penetrate membranes to reach intracellular targets, and unwanted immune responses. Thus, a strategy to turn inhibitory or neutralizing antibodies into small-molecule drugs, such as the one used to identify S2C3, could have a broad application, which is supported by the following theoretical considerations. First, interactions between an antibody and its cognate antigen involve hydrophobic interactions, hydrogen bonds, and salt bridges, similar to those between a small-molecule drug and its protein target. Second, protein–protein interactions often rely on a small set of contact residues (hot spot) for the majority of binding free energy despite large interfaces [157], suggesting that a small-molecule compound may be sufficient not only to mimic how an inhibitory antibody binds its antigen but also to compete with it for antigen binding. Third, effective antibodies often target functionally critical sites (inhibitory or neutralizing epitopes) on a protein of interest. Once a small-molecule lead is identified through competition with the antibody for antigen binding, it may mimic the action of the antibody to block or modulate physiological functions of the protein (antigen). When confirmed by functional and structural studies, it can serve as an excellent starting point for further drug development. Identification of the MPER-targeting fusion inhibitors is a proof-of-concept of this strategy.

## 5. Conclusions

Recent advances in structural biology have brought an unprecedented opportunity to study the HIV-1 entry process near or at the atomic resolution. New structures of HIV-1 Env and its cellular receptors not only continue to fill the major gaps in our knowledge on the mechanistic aspects but also open avenues for developing novel antiviral therapeutics.
